# Selective Binding of Cyclodextrins with Leflunomide and Its Pharmacologically Active Metabolite Teriflunomide

**DOI:** 10.3390/ijms21239102

**Published:** 2020-11-30

**Authors:** Irina Terekhova, Iliya Kritskiy, Mikhail Agafonov, Roman Kumeev, Carlos Martínez-Cortés, Horacio Pérez-Sánchez

**Affiliations:** 1 G.A. Krestov Institute of Solution Chemistry of Russian Academy of Sciences, 153045 Ivanovo, Russia; axon92@list.ru (I.K.); agafonov_m.a@mail.ru (M.A.); rsk@isc-ras.ru (R.K.); 2 Structural Bioinformatics and High Performance Computing Research Group (BIO-HPC), Universidad Católica de Murcia (UCAM), 30107 Guadalupe, Spain; cmartinez1@ucam.edu

**Keywords:** cyclodextrin, leflunomide, teriflunomide, complex formation, molecular modeling, selectivity

## Abstract

The selectivity of encapsulation of leflunomide and teriflunomide by native α-, β- and γ-cyclodextrins was investigated through ^1^H NMR and molecular modeling. Thermodynamic analysis revealed the main driving forces involved in the binding. For α-cyclodextrin, the partial encapsulation was obtained while deep penetration was characterized for the other two cyclodextrins, where the remaining polar fragment of the molecule is located outside the macrocyclic cavity. The interactions via hydrogen bonding are responsible for high negative enthalpy and entropy changes accompanying the complexation of cyclodextrins with teriflunomide. These results were in agreement with the molecular modeling calculations, which provide a clearer picture of the involved interactions at the atomic level.

## 1. Introduction

**Figure 1:**
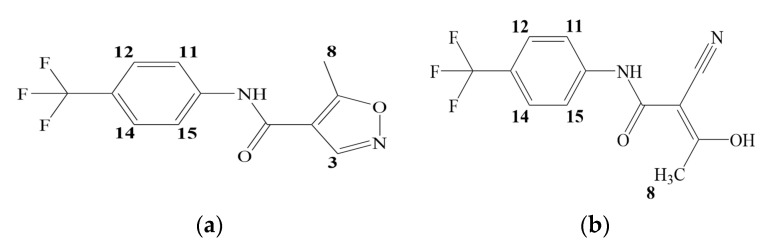
Structural formulas of leflunomide (**a**) and teriflunomide (**b**).

Leflunomide (LEF) is an isoxazole derivative (Figure 1), which displays anti-inflammatory and immunosuppressive properties and has been approved as a disease-modifying drug in the therapy of autoimmune diseases [1,2]. LEF is an oral prodrug, which is transformed during metabolism to its single active metabolite teriflunomide (TEF, Figure 1) [3]. Thus, the pharmacological activity of LEF is primarily mediated by TEF [4]. TEF has an open-ring structure with the same molecular formula and weight as LEF. Moreover, TEF was approved in 2012 for the treatment of relapsing forms of multiple sclerosis [5]. It has been indicated in several works [6,7] that LEF and TEF can display an anti-cancer effect.

In spite of high pharmacological activity, LEF and TEF are rather toxic [8]. In particular, the gastrointestinal side effects and liver injury limits the long-term application of these drugs. In this connection, different technologies have been used to improve pharmacologically important properties and to decrease the toxicity of LEF and TEF. For example, nanoliposomes were proposed as containers, which can effectively target and deliver the TEF at the site of inflammation [9,10]. Biodegradable microspheres containing polymers and LEF were prepared and efficiently used in intra-articular injections and oral dosage forms [11]. In our previous work [12], we used different polymers widely applied in the pharmaceutical industry with the purpose of increasing the aqueous solubility of the drug. The employment of a co-crystallization tool to enhance the solubility was evaluated [13,14]. Cadden et al. [13] demonstrated a 1.5-fold solubility increase for cocrystal of LEF with pyrogallol. A more pronounced rise (11 times) of solubility was detected for cocrystals of TEF with triethanolamine [14].

Cyclodextrins (CDs) are safe additives with widespread applications in different fields, including pharmaceuticals, biomedicine, and biotechnology [15,16]. Functional properties of CDs are determined by their ability to include complex formation with organic substrates. As it is well known [15,16,17,18], physicochemical properties and bioactivity of compounds included in CDs can be improved. In particular, solubility can be enhanced, and this can lead, on one hand, to a decrease in the dose of the drug and its toxicity, on the other hand, to an increase in the therapeutic effect of the drug.

The literature review showed that the complex formation of CDs was studied only with LEF [12,17]. It was found in our recent publication [12] that the solubilizing effect of native and modified β-cyclodextrins was more pronounced in comparison with α-CD and γ-CD. As it was demonstrated by Bankar and Mahatma [17], LEF forms an inclusion complex with hydroxypropylated β-CD both in an aqueous solution and solid-state. The stability constant of this inclusion complex is 224.3 M^−1^. Inclusion complexes of LEF with hydroxypropylated β-cyclodextrin obtained by freeze-drying method possessed increasing solubility and dissolution rate [17]. Recently, we tried to load LEF in the metal organic frameworks composed of potassium cations and γ-cyclodextrins as linkers [18]. A conversation of LEF in TEF during the loading into frameworks has been detected.

To the best of our knowledge, the complexation of CDs with TEF was not investigated, and this fact determines the novelty of the present research. Moreover, it was interesting to compare the binding affinity of CDs to prodrug (LEF) and its metabolite (TEF). Thus, the purpose of this work was to carry out the experimental and molecular modeling study on the interactions of native α-CD, β-CD, and γ-CD with LEF and TEF. Comparative analysis of the thermodynamics and binding mode of CDs with prodrug LEF and its metabolite TEF was performed. It was interesting to reveal the selectivity of binding and the role of structural factors (size of the macrocyclic cavity and opening isoxazole ring) in the complexation process.

## 2. Results and Discussion

### 2.1. Experimental Study of Complex Formation of CDs with LEF and TEF

As it is well known [2,4,5,6], TEF is the pharmacologically active metabolite of LEF. Moreover, TEF is a relatively new innovative drug applied for multiple sclerosis treatment [5]. TEF can be obtained from LEF through the opening of the isoxazole ring (Figure 1). In this connection, it would be interesting to reveal the influence of the isoxazole ring transformation on the binding affinity of these two compounds to CDs. To this end, the binding of native CDs with LEF and TEF was studied by experimental (^1^H NMR) and molecular modeling methods.

^1^H NMR spectra of LEF and TEF were recorded in the presence of variable amounts of α-CD, β-CD and γ-CDs. Chemical shift changes (Δ*δ*) of the protons of LEF and TEF were calculated as follows:

(1) $$\Delta \delta =\delta_{complexed}-\delta_{free}$$

where *δ_complexed_* and *δ_free_* are chemical shifts of the drugs in complexed and free states, respectively.

The dependencies of the chemical shift changes versus CD concentration are given in Figure S1. These concentration dependences were used for calculation of the binding constants (*K*) and chemical shift changes induced by 100% complex formation (Δ*_c_δ*). The fitting procedure was based on the 1:1 binding mode, which was confirmed by Job’s method [19]. Job plots (Figure S2) displayed extremes at molar fraction corresponding to 1:1 complex formation in all systems under study.

To obtain thermodynamic parameters of complex formation, the ^1^H NMR experiments were carried out in the temperature range from 288.15 K to 318.15 K. The enthalpy and entropy changes of complex formation (Δ*_c_H* and Δ*_c_S*) were derived from the temperature dependence of the binding constant using van’t Hoff approach:

(2) $$\ln K=-\frac{\Delta_{c}H}{RT}+\frac{\Delta_{c}S}{R}$$

where *R* is the gas constant; *T* is temperature. The dependencies of *lnK* versus *1/T* are shown in Figure S3. Values of Δ*_c_δ* and thermodynamic parameters of complex formation (*K*, Δ*_c_G,* Δ*_c_H* and Δ*_c_S*) are summarized in Table 1 and Table 2, respectively. It should be mentioned herein that the complex formation of CDs with TEF was studied in phosphate buffer (pH 7.4). The choice of this buffer was determined by the very low solubility of TEF in water and in the acidic medium [20]. In this case, the ionization state of TEF should be taken into account since the TEF molecule has the ionizable functional groups –NH (*pK*_1_ = 5.4) and –OH (*pK*_1_ = 10.4) [21]. The distribution of different ionized forms of TEF depending on pH (Figure S4) shows that TEF anionic species are predominant at pH 7.4. On the contrary, the binding of CDs with LEF was investigated in water. We did not use the phosphate buffer for these systems in order to avoid the LEF → TEF transformation. As it has been documented [22], LEF can be transformed into TEF in the alkaline medium under the action of –OH groups as a catalyst. The LEF is ionized at pH > 9 [23]; therefore, in water, it exists as an uncharged molecule.

In the ^1^H NMR spectrum of LEF (Figure S4a), the signals of the protons of isoxazole ring (H_3_), benzene ring (H_11_, H_12_, H_14_ и H_15_) and methyl group (H_8_) are visible. In the ^1^H NMR spectrum of TEF (Figure S4b), there are the signals of the protons on benzene ring (H_11_, H_12_, H_14_ и H_15_) and methyl group (H_8_). Analysis of Δ*_c_δ* values reported in Table 1 allows to propose the binding mode.

**Figure 2:**
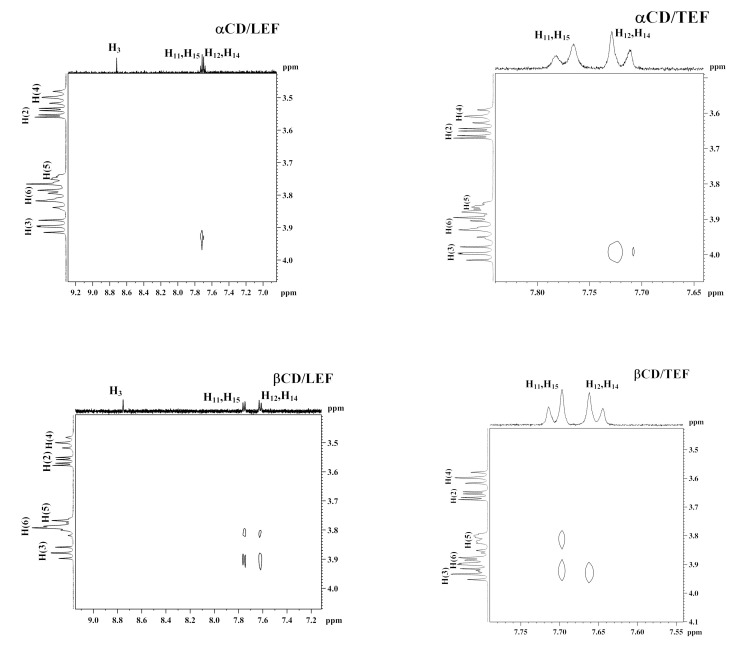

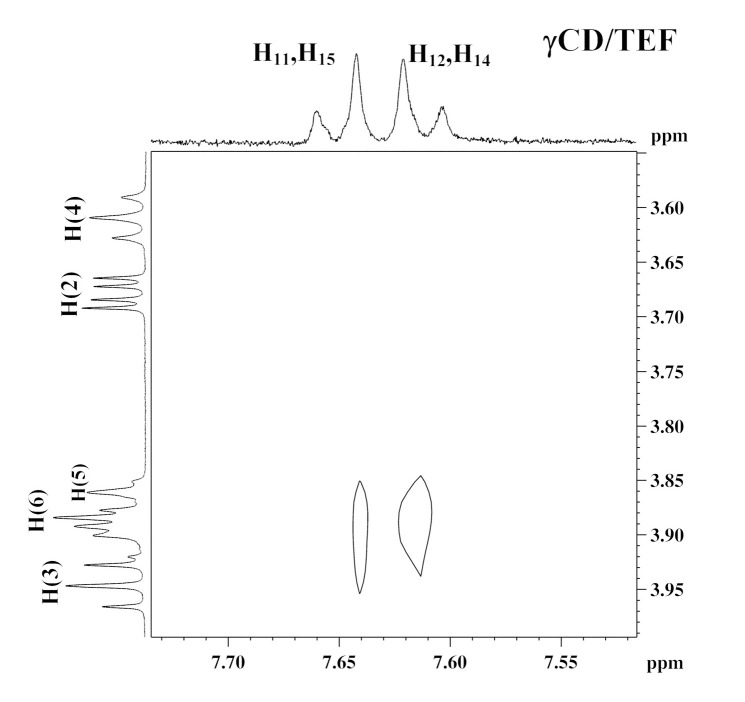
2D ^1^H NMR ROESY spectra of complexes of CDs with LEF and TEF.

For the complex formation of α-CD with LEF, the signals of H_12_ and H_14_ protons were significantly shifted (Table 1). Downfield shits of these protons can be caused by the inclusion of benzene ring into the hydrophobic cavity of α-CD and its location in a less polar environment compared with water. The Δ*_c_δ* for H_3_ was also measurable (Table 1), and it can be due to the insertion of LEF isoxazole ring into α-CD cavity or its participation in the surface interactions with hydroxyls surrounding the α-CD cavity. To give insight into the binding mode of α-CD with LEF, the ROESY spectrum was recorded and analyzed (Figure 2). As one can see from Figure 2, there is only one cross peak between protons of LEF benzene ring and H(3) protons of α-CD. It should be mentioned herein that CD’s protons H(3) and H(5) located inside the macrocyclic cavity are sensitive to the inclusion of guest molecules [24]. Unfortunately, it was not possible to analyze the induced by complex formation chemical shift changes of α-CD protons due to a very low concentration of LEF and weak binding in this system. Thus, the obtained results point out the partial inclusion of LEF molecule into α-CD cavity. Only the benzene ring is partially inserted into the cavity, and the isoxazole ring is outside and interacts with the external –OH groups of α-CD.

As it is well known [15,16], inclusion complexes of CDs are formed via noncovalent interactions such as Van der Waals and hydrophobic interactions, H-bonding, and, etc. Complex formation of α-CD with LEF is characterized by moderate negative Δ*_c_H* and small positive *T*Δ*_c_S* values (Table 2). Hydrophobic interactions of the LEF aromatic ring with α-CD cavity as well as dehydration of both reagents are responsible for the positive entropy changes. Complexes α-CD/LEF are characterized by low stability, and they are mainly enthalpy stabilized.

**Figure 3:**
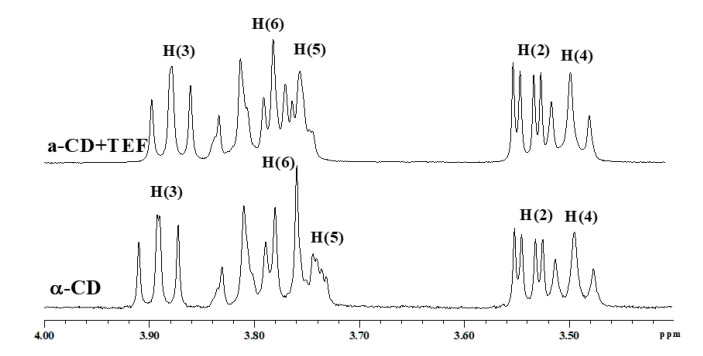

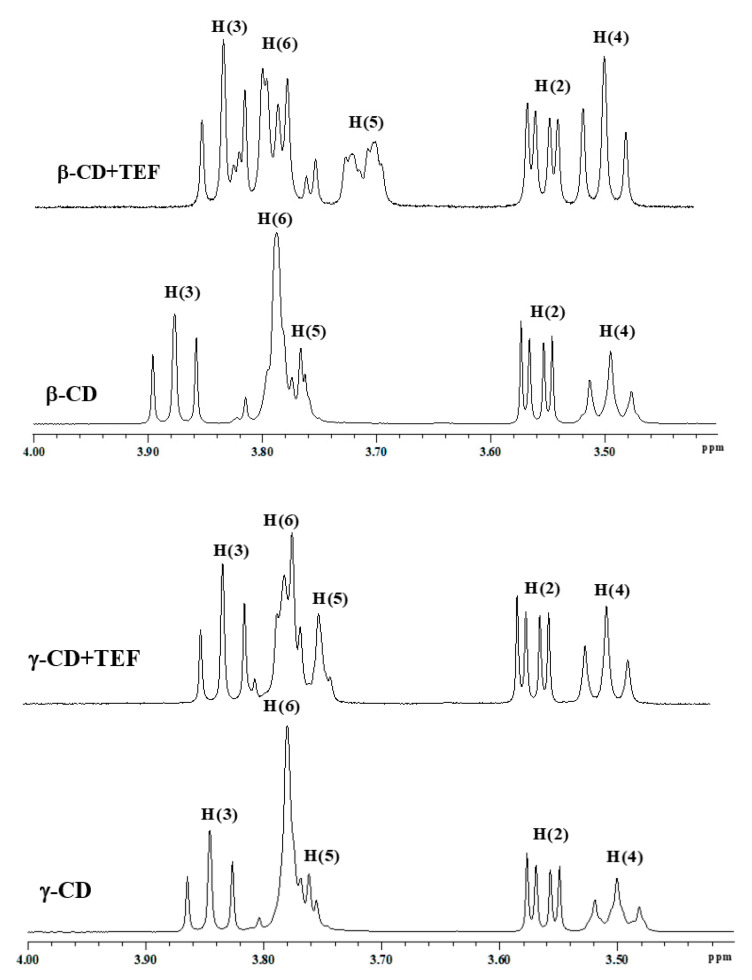
Fragments of ^1^H NMR spectra of CDs with and without TEF in phosphate buffer (pD 7.4) at 298.15 K.

On the contrary, complex formation of α-CD with TEF is accompanied by high negative Δ*_c_H* and *T*Δ*_c_S* (Table 2). Thus, the opening of the isoxazole ring during LEF → TEF transformation considerably changes the thermodynamics of binding with α-CD. As follows from the ^1^H NMR data (Table 1), the benzene ring is inserted into the α-CD cavity while the remaining part of the TEF molecule is placed outside and can interact with CD’s hydroxyls. More probably, this inclusion is partial since α-CD consisting of 6 glucose units has the smallest cavity among the native CDs under study. Depth of the penetration into the cavity can be estimated based on the analysis of the ^1^H NMR spectrum of α-CD and ROYSY spectrum. Figure 3 shows fragments of ^1^H NMR spectra of α-CD with and without TEF. A noticeable shifting observed only for H(3) protons points out the shallow inclusion of TEF into α-CD cavity. ROESY spectrum also displays cross-peaks only between α-CD proton H(3) and protons of the TEF benzene ring (Figure 2). According to the proposed binding mode, TEF polar groups that remained outside the cavity can form H-bonds with the external α-CD hydroxyls. Namely, rather polar –C≡N group and other polar groups of TEF such as –C=O and –OH as well as the ionized –NH group participate in the hydrogen bonding with α-CD hydroxyls surrounding the macrocyclic cavity. As a result, complex formation of α-CD with TEF is highly exothermic, and inclusion complexes are more structured (*T*Δ*_c_S<<0)*.

For the complex formation of β-CD with LEF and TEF, a similar trend of the chemical shift changes was observed (Table 1). The signals of H_11_ and H_15_ protons are downfield shifted, while the signals of H_12_ and H_14_ protons are upfield shifted. The molecular cavity of β-CD composed of seven glucose units is larger compared with α-CD and, therefore, deeper inclusion of LEF and TEF is possible. As one can see from Figure 3, signals of the inner H(3) and H(5) protons are upfield shifted. The ROESY spectra (Figure 2) also show two cross-peaks between protons of the benzene ring of LEF and TEF and the inner protons of β-CD. This is typical for the deep penetration of the benzene ring into the cavity.

Deep insertion of the benzene ring of LEF and TEF induces extensive dehydration of β-CD cavity as well as hydrophobic interactions. As a result, the increased positive contribution from these two processes to Δ*_c_H* and *T*Δ*_c_S* is observed. Compared with α-CD, complex formation of drugs with β-CD is characterized by less negative Δ*_c_H* and more positive *T*Δ*_c_S* (Table 2). Complexes β-CD/LEF are enthalpy–entropy stabilized, with entropy contribution being predominant. Similar to α-CD, complexation of β-CD with TEF is more energetically favorable than with LEF. This fact is determined by the hydrogen bonding of the polar side group of TEF with CD hydroxyls. Complexes β-CD/TEF are mainly enthalpy stabilized (Table 2).

The important role of TEF polar side groups in complex formation is further evidence for the binding with γ-CD. The molecular cavity of γ-CD consisting of eight glucose units is bulkier. It can accommodate more than one drug molecule. However, Job plots (Figure S2) demonstrate a 1:1 complex formation of γ-CD with TEF and LEF. According to the 1:1 binding mode, strong retention of the drug molecules inside γ-CD cavity does not occur due to an absence of close contact between the interacting sites. Consequently, the γ-CD complexes should not be stable. This is really true for γ-CD/LEF complexes (Table 2). As one can see from the thermodynamic parameters, binding of γ-CD with LEF is characterized by the moderate negative Δ*_c_H* and moderate positive *T*Δ*_c_S* (Table 2). It was not possible to obtain a 2D ROESY spectrum for this system. On the contrary, the interaction of γ-CD with LEF is highly exothermic and accompanied by the formation of rather stable complexes. We suppose that participation of the polar moiety of TEF molecule in the hydrogen bonding with –OH groups of γ-CD is the main source of high negative values of Δ*_c_H* and *T*Δ*_c_S*. The location of the benzene ring in the macrocyclic cavity is confirmed by the considerable shifting of the aromatic protons H_11_, H_12_, H_14_ and H_15_ of TEF (Table 1) and the internal H(3) and H(5) protons of γ-CD (Figure 3). Deep insertion of TEF benzene ring into γ-CD cavity is confirmed by the availability of cross-peaks between the aromatic protons of the guest and H(3) and H(5) protons of the host in the ROESY spectrum (Figure 2).

**Figure 4:**
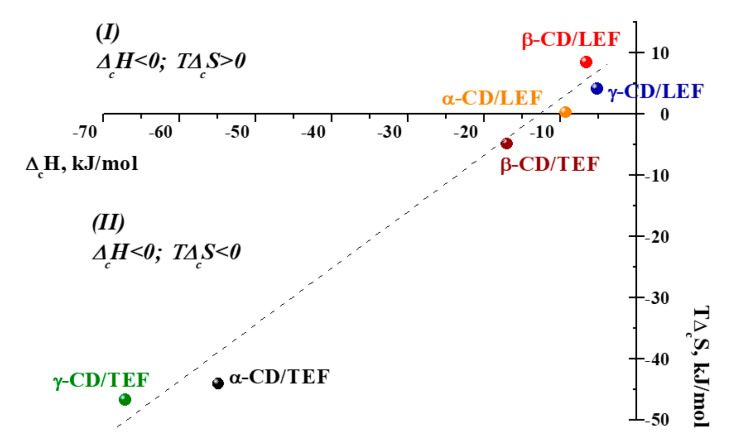
Enthalpy–entropy compensation for complex formation of CDs with LEF and TEF at 298.15 K.

It is interesting to consider the phenomenon of enthalpy–entropy compensation in the reactions of the complex formation of CDs with LEF and TEF. Generally, a favorable enthalpy of binding is accompanied by the unfavorable entropy change due to a decrease of the system configuration freedom upon complex formation [25]. Dependence of *T*Δ*_c_S* on Δ*_c_H* is shown in Figure 4. It seems that a linear correlation is observed. However, the complexes formed by LEF and TEF have different positions on the graph. Complexes of CDs with LEF belong to the I group, which is characterized by negative enthalpy changes and positive entropy changes. Complexes of CDs with TEF correspond to II group with negative Δ*_c_H* and negative *T*Δ*_c_S*. Thus, CD/LEF complexes are enthalpy–entropy stabilized due to the prevalence of hydrophobic interactions and dehydration, which are realized when the aromatic ring of the gusts is included in the macrocyclic cavity of the host. Complexes of CD/TEF are only enthalpy stabilized. The formation of these complexes is governed by the participation of the polar part of the TEF molecule in the hydrogen bonding with the external hydroxyls of CDs, with the TEF benzene ring penetrating the inner CD cavity. In this case, binding is energetically favorable and results in the formation of more structured complexes.

In conclusion, the experimental study of complex formation showed the difference in the driving forces of CD binding with LEF and TEF. The key role of the polar side groups of TEF in the binding with CDs was proposed on the basis of the comparative analysis of the thermodynamics of complex formation. Unfortunately, the participation of these groups in the hydrogen bonding with CDs could not be confirmed by ^1^H NMR experiments. However, theoretical calculations can provide insights about the interactions of CDs with LEF and TEF at the atomic level and can be applied to our case.

### 2.2. Molecular Modeling of Complex Formation of CDs with LEF and TEF

**Figure 5:**
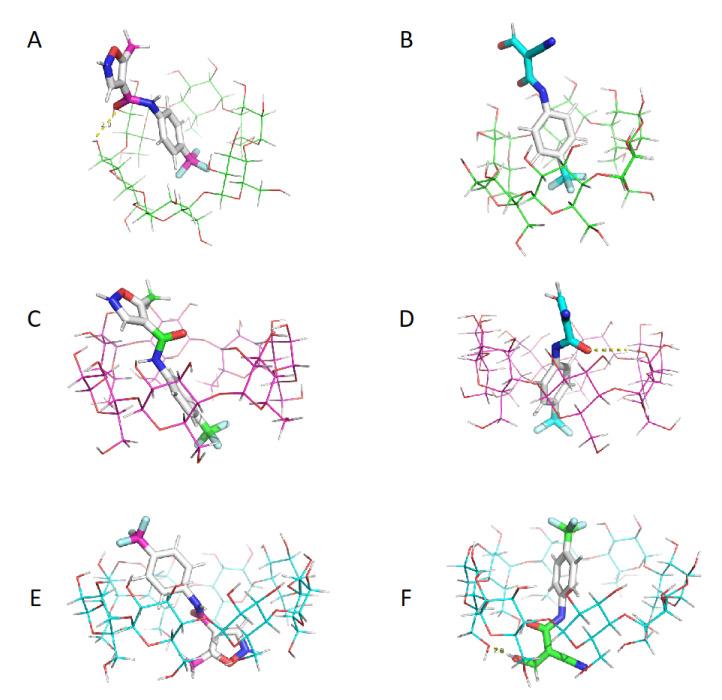
Docking results obtained for (**A**) α-CD/LEF, (**B**) α-CD/TEF, (**C**) β-CD/LEF, (**D**) β-CD/TEF, (**E**) γ-CD/LEF and (**F**) γ-CD/TEF. Cyclodextrin core is represented with thin lines and hydrogen bonds with yellow dashes.

Main structural results obtained for the molecular modeling of complex formation of CDs with LEF and TEF can be shown in Figure 5. First, we can observe the partial complexation of LEF and TEF with α-CD, and the deeper penetration of LEF and TEF with both β-CD and γ-CD. In Figure 5A,D,F, the formation of hydrogen bonds between oxygens from the polar part of LEF or TEF and hydroxyl groups from CDs are obtained, in agreement with the thermodynamic data (Table 2) and ^1^H NMR results (Table 1, Figure 3). We can also observe that obtained free energy estimations by MMGBSA are in qualitative agreement with experimental values.

## 3. Materials and Methods

### 3.1. Materials

Leflunomide, teriflunomide, α-, β- and γ-cyclodextrins were purchased from Sigma-Aldrich. All other reagents (HCl, KH_2_PO_4_ and Na_2_HPO_4_⋅12H_2_O) used for the preparation of the buffers were of analytical grade. The pH of buffer solutions was controlled using Mettler Toledo Five Easy pH-meter.

### 3.2. ^1^H NMR Spectroscopy

The ^1^H NMR spectra were recorded by using a Bruker-AV-500 spectrometer. Measurements were performed at a constant temperature of 298.15 K, maintained with the help of a Bruker BVT-3000 temperature controller. Cyclohexane was employed as an internal reference.

^1^H NMR spectra of LEF were recorded in deuterated water (isotopic purity is 99.9%). In the case of TEF, phosphate buffer (pH 7.4) prepared on the basis of deuterated water was used since TEF is poorly soluble in water [20].

### 3.3. Job’s Method

Job’s method was used for the determination of the complex stoichiometry [19]. According to this method, the total molar concentration of CD and drug was held constant, but their mole fractions were varied. Job plots were constructed by plotting the chemical shift change (Δ*δ*) against the molar fraction (*R*) of the drug in the binary mixture with CD. The extreme of the Job plot indicates the complex stoichiometry.

### 3.4. Molecular Modeling

The molecular structures for LEF and TEF used in this study were built manually using AutoDock Tools [26] and structural information derived from experimental data. Regarding LEF, only Z isomer was considered due to its higher stability [27]. Dissociation of TEF has 2 stages (pK_a1_ = 5.2, pK_a2_ = 10.4 [21]), so we considered the monoanion with the -NH group ionized. The structures of α-CD, β-CD and γ-CD were extracted from the crystal structures of the Protein Data Bank (PDB) with codes 2ZYM, 2ZYN and 2ZYK and post-processed with AutoDockTools [26]. Molecular docking calculations were carried out using default parameters in AutoDock Vina [28]. Graphical representations of the docking results were prepared using PyMOL (version 2.4.1, Schrödinger, LLC).

The molecular mechanical/generalized Born surface area (MMGBSA) method was applied in order to estimate the free energy of binding with more accuracy than Autodock’s scoring function, using the Prime module of the Maestro Suite 2019.3 (www.schroedinger.com).

## 4. Conclusions

The binding affinity of native α-CD, β-CD and γ-CD to LEF and its metabolite TEF was investigated using ^1^H NMR and molecular modeling. Comparative analysis was carried out in terms of the influence of the structural factor on the thermodynamics and binding mode. It was demonstrated that the inclusion of LEF and TEF in the smallest α-CD cavity is partial, whereas the deep penetration into the molecular cavity of β-CD and γ-CD takes place. The inclusion of the LEF benzene ring is more preferred compared with the LEF isoxazole ring. Benzene ring of TEF is placed inside the macrocyclic cavity, and the remaining polar fragment of the TEF molecule is located outside and interacts with the external hydroxyls of CD. These interactions via hydrogen bonding are responsible for high negative enthalpy and entropy changes accompanying the complexation of CDs with TEF. These results were in agreement with reported molecular modeling calculations, which provide a clearer picture of the involved interactions at the atomic level.

## Figures and Tables

**Table 1:** Chemical shift changes (Δ*_c_δ*, ppm *) of protons of leflunomide (LEF) and teriflunomide (TEF) induced by complex formation with cyclodextrins (CDs) at 298.15 K **.

CD	LEF				TEF		
	H_3_	H_8_	H_11_, H_15_	H_12_, H_14_	H_8_	H_11_, H_15_	H_12_, H_14_
α-CD	0.09	0.04	0.08	0.17	<0.01	0.56	−0.02
β-CD	0.08	0.04	0.18	−0.14	0.01	0.02	−0.06
γ-CD	0.04	0.05	−0.02	0.08	<0.01	−0.18	−0.16

*—error of Δ*_c_δ* determination was 0.01 ppm; **—in deuterated water for LEF and deuterated phosphate buffer (pH 7.4) for TEF.

**Table 2:** Thermodynamic parameters of complex formation of CDs with LEF and TEF * and free energy estimation by molecular mechanical/generalized Born surface area (MMGBSA).

Complex	K	Δ_c_G,	Δ_c_H,	TΔ_c_S,	Δ_c_G MMGBSA
		kJ/mol	kJ/mol	kJ/mol	kJ/mol
α-CD/LEF	49 ± 3	−9.6 ± 0.9	−9.3 ± 1.4	0.3 ± 0.1	−82
	58 ± 2 **	−10.1 ± 0.4	‒	‒	‒
β-CD/LEF	446 ± 30	−15.1 ± 1.5	−6.6 ± 1.0	8.5 ± 1.7	−117.8
	390 ± 20 **				
γ-CD/LEF	44 ± 3	−9.4 ± 0.9	−5.2 ± 0.8	4.2 ± 0.8	−103.5
	100 ± 2 **				
α-CD/TEF	79 ± 6	−11 ± 1	−55 ± 4	−44 ± 8	−145
β-CD/TEF	138 ± 10	−12 ± 1	−17 ± 2	−5 ± 1	−114.2
γ-CD/TEF	3722 ± 295	−20 ± 2	−67 ± 5	−47 ± 9	−143.1

*—in deuterated water for LEF and in deuterated phosphate buffer for TEF; **—early obtained by UV-vis spectroscopy at 22 °C [12].

## Supplementary Materials

Supplementary materials can be found at https://www.mdpi.com/1422-0067/21/23/9102/s1. Figure S1: Dependences of chemical shift changes on CD concentration at 25 °C (a—α-CD/LEF, b—α-CD/TEF, c—β-CD/LEF, d—β-CD/TEF, e—γ-CD/LEF, f—γ-CD/TEF); Figure S2: Job plots for complex formation of CDs with LEF and TEF; Figure S3: van’t Hoff dependences for complex formation of CDs with LEF and TEF; Figure S4: Distribution of different forms of TEF depending on pH.

## Abbreviations

CD	Cyclodextrin
LEF	Leflunomide
TEF	Teriflunomide
