# Supplementary material for: Selective Binding of Cyclodextrins with Leflunomide and Its Pharmacologically Active Metabolite Teriflunomide
Source: Int J Mol Sci. 2020 Nov 30;21(23):9102. doi: 10.3390/ijms21239102 (PMC7730839; doi:10.3390/ijms21239102)
Supplement: Supplementary file 1 [file ijms-21-09102-s001.pdf]

Supporting Information

Different complexation affinity of native cyclodextrins to leflunomide and its pharmacologically active metabolite teriflunomide

Irina Terekhova[1,*], Iliya Kritskiy [1], Mikhail Agafonov[1], Roman Kumeev [1], Carlos Cortes-Martínez and Horacio Pérez-Sánchez[2,*]

1 G.A. Krestov Institute of Solution Chemistry of Russian Academy of Sciences, Ivanovo, ivt@isc-ras.ru
2 Structural Bioinformatics and High Performance Computing Research Group (BIO-HPC), Universidad Católica de Murcia (UCAM), Guadalupe, Spain ; hperez@ucam.edu (H.P.-S.) ; cmartinez1@ucam.edu (C.C.-M.)
* Correspondence: ivt@isc-ras.ru, hperez@ucam.edu

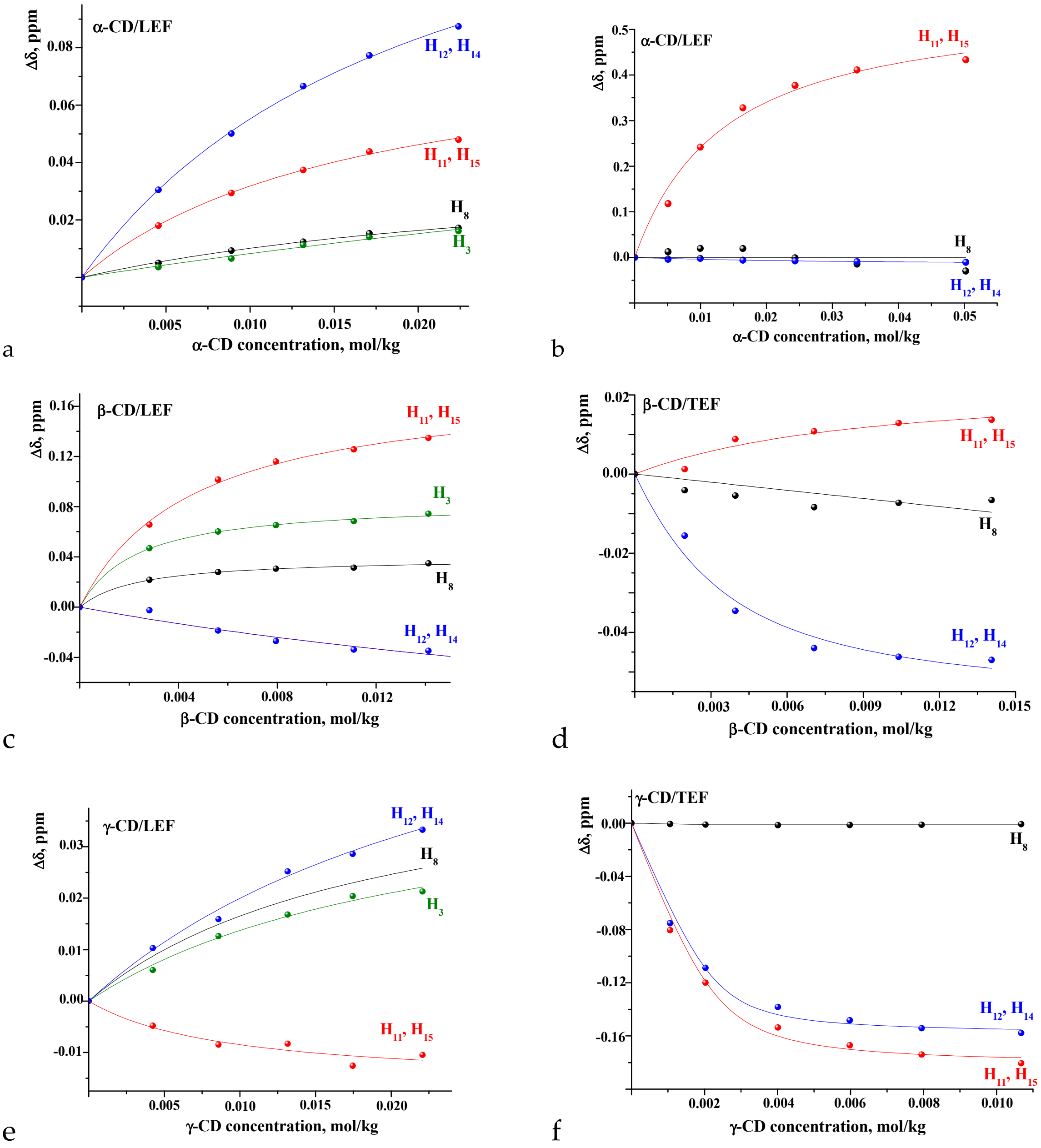

Figure S1. Dependences of chemical shift changes on CD concentration at 25 °C.

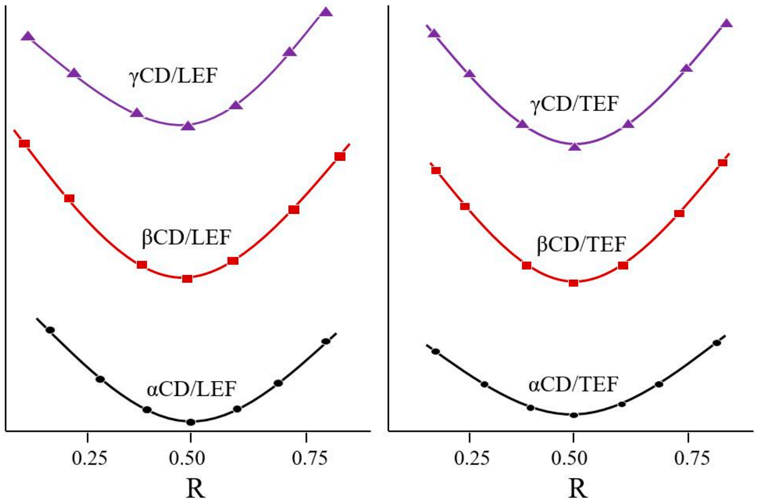

Figure S2. Job plots for complex formation of CDs with LEF and TEF.

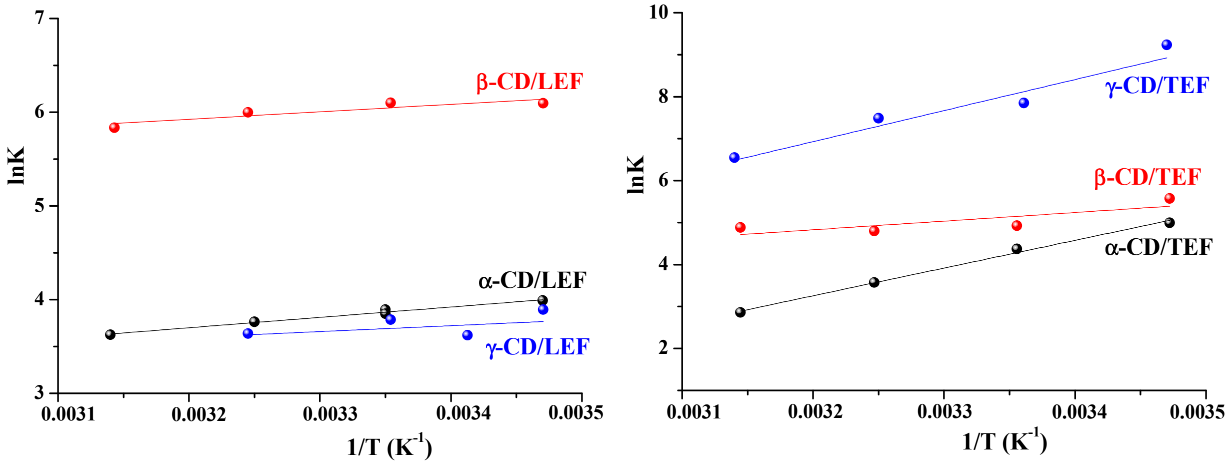

Figure S3. van’t Hoff dependences for complex formation of CDs with LEF and TEF.

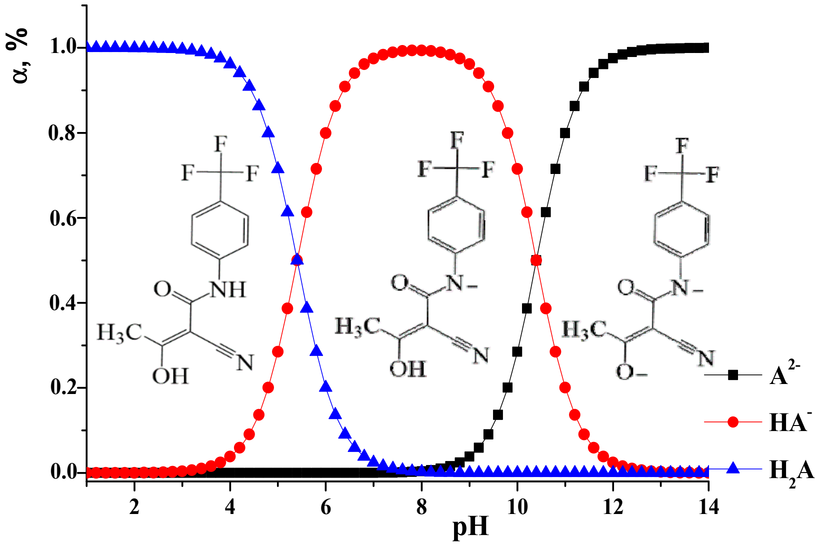

Figure S4. Distribution of different forms of TEF depending on pH.
